# ECG-Free Heartbeat Detection in Seismocardiography Signals via Template Matching

**DOI:** 10.3390/s23104684

**Published:** 2023-05-12

**Authors:** Jessica Centracchio, Salvatore Parlato, Daniele Esposito, Paolo Bifulco, Emilio Andreozzi

**Affiliations:** Department of Electrical Engineering and Information Technologies, University of Naples Federico II, Via Claudio 21, 80125 Naples, Italy; jessica.centracchio@unina.it (J.C.); sal.parlato@studenti.unina.it (S.P.); daniele.esposito@unina.it (D.E.); paolo.bifulco@unina.it (P.B.)

**Keywords:** seismocardiography, heartbeat detection, template matching, heart rate, mechanocardiography

## Abstract

Cardiac monitoring can be performed by means of an accelerometer attached to a subject’s chest, which produces the Seismocardiography (SCG) signal. Detection of SCG heartbeats is commonly carried out by taking advantage of a simultaneous electrocardiogram (ECG). SCG-based long-term monitoring would certainly be less obtrusive and easier to implement without an ECG. Few studies have addressed this issue using a variety of complex approaches. This study proposes a novel approach to ECG-free heartbeat detection in SCG signals via template matching, based on normalized cross-correlation as heartbeats similarity measure. The algorithm was tested on the SCG signals acquired from 77 patients with valvular heart diseases, available from a public database. The performance of the proposed approach was assessed in terms of sensitivity and positive predictive value (PPV) of the heartbeat detection and accuracy of inter-beat intervals measurement. Sensitivity and PPV of 96% and 97%, respectively, were obtained by considering templates that included both systolic and diastolic complexes. Regression, correlation, and Bland–Altman analyses carried out on inter-beat intervals reported slope and intercept of 0.997 and 2.8 ms (R^2^ > 0.999), as well as non-significant bias and limits of agreement of ±7.8 ms. The results are comparable or superior to those achieved by far more complex algorithms, also based on artificial intelligence. The low computational burden of the proposed approach makes it suitable for direct implementation in wearable devices.

## 1. Introduction

Continuous heart rate monitoring is fundamental in critical medical circumstances to assess cardiac function. It is a vital task in intensive care units, surgery, post-anesthesia surveillance, and emergency medicine [1,2,3]. Heart rate monitors are extensively used for analysis of heart rate variability (HRV), which is considered a strong predictor of the outcome in patients with high cardiovascular risk [4,5]. Indeed, people with coronary artery disease, heart failure, valvular heart disease, or after a myocardial infarction, ischemia, or a stroke need to be continuously monitored to provide them with timely therapeutic intervention, thus preventing the deterioration of health conditions and the occurrence of fatal arrhythmic events [1,4,6]. HRV analysis has also been recognized as a valuable non-invasive tool for identifying cardiac autonomic dysfunction in neurological (e.g., multiple sclerosis), renal (e.g., end-stage renal failure), or metabolic (e.g., diabetes, obesity) diseases [6,7,8,9,10]. Moreover, heart rate and HRV indices can be useful: in sleep medicine to assess sleep quality and detect sleep apnea syndrome, or neonatal distress syndrome [1,6,8,11]; in fitness and sport science to monitor exercise training and the performance of athletes [8,12,13]; or even in rehabilitation medicine to track the exercise of chronic patients [1,8]. Furthermore, they can provide valuable information: to evaluate the effect of drugs [8]; to monitor stress levels and obtain clear evidence of emotion disorders (e.g., anxiety, depression, etc.) [1,14]; to promote lifestyle awareness in people and improve their well-being [1,8]; and to enhance road safety by detecting driver fatigue [15].

Electrocardiography (ECG) records the electrical activity produced by the heart on the body’s surface and is currently regarded as the gold standard for heart rate monitoring in clinical routine. Heart rate measurements rely on heartbeat detection, which is usually accomplished by locating the R-wave of each QRS complex in ECG signals [1,16,17,18,19,20,21]. The well-known Pan and Tompkins algorithm is the most popular approach for this purpose [1,22]. However, the ECG has some drawbacks: it is intrinsically affected by electromagnetic interference, needs stable placement of the electrodes over time (electrode slipping and detachment may happen) and good skin–electrode impedance (which worsens with the drying of the electrolytic gel), involves electrical risks. In addition, it must be performed by skilled clinical professionals and is somewhat uncomfortable. Therefore, this technique is not well suited to continuous, long-term monitoring, especially in daily life environments, although some portable instruments, such as the Holter device, have been proposed as possible solutions for home monitoring (the Holter monitor generally allows recordings of no longer than 72 h). Furthermore, the ECG does not provide any information about the cardiac mechanical function [1,16,17,18,19,20,21].

In addition to the ECG, heart rate monitoring can also be pursued by detecting the peripheral effect of cardiac contractions through the Photoplethysmography (PPG) technique. The PPG signal is a non-invasive recording of blood volume changes in a macrovascular bed of the skin due to arterial pulsations. Particularly, blood volume variations are monitored indirectly by measuring changes in the light absorption or transmission of human tissues at characteristic wavelengths. Generally, beat-by-beat heart rate measurement via PPG involves identifying the temporal locations of specific markers on the signal waveform, such as the systolic peak, which are related to important cardiac cycle events. PPG sensors are also integrated into wearable devices (e.g., smartwatches). However, despite the simplicity, low-cost, and unobtrusiveness of this technique, PPG signal quality is very sensitive to motion artifacts, which impair accurate systolic peak detection. Data corrupted by motion artifacts are often unusable, thus jeopardizing continuous heart rate monitoring [1,23,24,25,26,27,28].

Since the second half of the 19th century, a variety of non-invasive, cardio-mechanical monitoring tools have been proposed to record the small vibrations generated by the mechanical activity of the beating heart and to obtain a more comprehensive evaluation of cardiac function. Among these, Ballistocardiography (BCG) [29,30,31,32,33,34], Phonocardiography (PCG) [35,36,37,38], and Seismocardiography (SCG) [39,40,41,42,43,44,45,46] are the subjects of current research. On one hand, BCG records whole-body vibrations due to the blood flowing through the vascular system by means of different instruments, such as weighing scales, systems embedded in a bed or chair, and even wearable sensors, which are particularly appealing for continuous monitoring applications. On the other hand, PCG captures the sonic components of cardiac-induced mechanical vibrations of the thorax, commonly known as heart sounds, by using modern electronic stethoscopes, while SCG measures infrasonic precordial accelerations. Specifically, the availability of lightweight, miniaturized, low-cost accelerometers, manufactured via micro-electromechanical systems (MEMS) technologies, has led SCG to gain particular attention, especially for the development of wearable devices. In recent years, novel, non-invasive cardio-mechanical monitoring techniques have been introduced, namely Gyrocardiography (GCG) [47,48], Kinocardiography (KCG) [49,50], and Forcecardiography (FCG) [51,52,53,54,55,56,57]. GCG records the three-dimensional angular velocities of the precordium via gyroscopes, which are often integrated into inertial measurement units (IMUs) that also contain accelerometers. For this reason, the combined use of GCG and SCG has also been investigated [58]. KCG, instead, results from the simultaneous acquisition of BCG and SCG signals. Finally, FCG measures the local, cardiac-induced mechanical vibrations of the chest wall via piezoresistive or piezoelectric force sensors. Particularly, FCG sensors are characterized by a very broad bandwidth, which allows them to monitor respiration, ventricular volume variations, an SCG-like component, and heart sounds, all simultaneously from a single contact point on the chest.

Measurement of the instantaneous heart rate by cardio-mechanical monitoring techniques is based on beat-by-beat localization of important cardiac cycle events (e.g., the heart valves opening and closing, blood ejection, isovolumic contraction, cardiac filling, etc.), which are marked by specific peaks and valleys. This allows for the estimation not only of the inter-beat intervals (or the instantaneous heart rate), but also further cardiac time intervals of clinical relevance, such as the pre-ejection period, the left ventricular ejection time, and the total systolic time [44,45,54,58,59]. Heartbeat localization incardio-mechanical signals is generally performed by assuming the ECG as a temporal reference, particularly the R-wave in each cardiac cycle. However, research is currently proceeding towards the development of automated, standalone (i.e., ECG-independent) methods, most of which rely on SCG signals. SCG-based heartbeat detectors exploit different approaches, such as signal envelope extraction, often combined with a thresholding operation [60,61,62,63,64,65,66,67,68,69]; continuous or discrete wavelet transform [70,71]; variational mode decomposition [72]; autocorrelated differential algorithm [58]; matched filtering [73]; probabilistic methods, e.g., the hidden Markov model [74]; machine learning [75]; and deep learning [76,77]. Of these, the heartbeat detection methods reported in [58,63] were performed on SCG acquisitions combined with simultaneous GCG recordings. It is important to note that the heartbeat detectors mentioned above, especially those based on artificial intelligence algorithms, have a rather high computational complexity. It is also worth noting that they were tested exclusively on signals from healthy subjects, except for [63], in which 12 patients with coronary artery disease were also considered. This aspect is important because cardiac diseases cause dysfunctions in the mechanical behavior of the beating heart, which result in morphological changes and/or instabilities in the cardio-mechanical signals that may impair the performance of heartbeat detection algorithms. Indeed, in [78], the authors analyzed the waveforms of SCG signals acquired from 90 pathological subjects with myocardial infarction, heart failure, or a transplanted heart, and found that in 38% of subjects the SCG waveform and fiducial points were altered with respect to the traditional references reported in the literature for healthy subjects.

Recently, some authors have presented a template matching approach for ECG-free heartbeat detection in the FCG signal, along with preliminary results on a small cohort of healthy subjects [79]. In particular, it has been applied to the high-frequency component of the FCG signal, which has been shown to share a very high similarity with the SCG signal [51,52,54].

This study was aimed at verifying the feasibility of this approach for SCG signals and achieving an extensive estimation of its performance on a much larger cohort of pathological subjects. Template matching based on normalized cross-correlation is a well-known technique for event detection, and its application to SCG signals could provide a simple, yet effective and robust method for ECG-free heartbeat detection. Indeed, the template matching approach has an intrinsic ability to deal with the inter-subject variability in SCG morphology, because it does not rely on any a priori assumption about the signal shape or specific fiducial points to recognize the signal chunks that match the selected template. Methods based on template matching have previously been applied to BCG [80] and PCG [81] signals, but they have been assessed only on a very small cohort of healthy subjects. The proposed template matching approach, instead, was tested on a publicly available database of SCG signals acquired from patients with valvular heart diseases (VHDs). To the best of our knowledge, no heartbeat detection method reported in the literature has been proven on such a large cohort of pathological subjects. The beat-by-beat performance of the proposed algorithm was assessed by comparing the inter-beat interval estimates obtained from the SCG signals with those obtained from simultaneous ECG recordings. The results of the statistical analyses carried out to compare the SCG- and ECG-based measures demonstrate that the proposed approach can provide very accurate measurements of inter-beat intervals, even on a large cohort of patients affected by one or more VHDs.

## 2. Materials and Methods

### 2.1. Dataset

In this study, SCG signals from a publicly available database [82] were analyzed. The database includes SCG recordings from 100 patients (59 males and 41 females, age 68 ± 14 years) with VHDs. The data were collected from two medical centers: 70% in China (patient IDs #CP-01 to #CP-70) and 30% in the United States (patient IDs #UP-01 to #UP-30). All subjects were diagnosed with one or more VHD conditions, namely moderate or greater aortic valve stenosis, aortic valve regurgitation, mitral valve stenosis, mitral valve regurgitation, and tricuspid valve regurgitation. The database also contains ECG signals, which were assumed as the ground truth for heartbeat detection.

Simultaneous SCG and ECG recordings were obtained from a three-dimensional MEMS accelerometer and a biopotential circuit, respectively. Both sensors were embedded in the Shimmer 3 ECG module (Shimmer Sensing, Dublin, Ireland). The device was firmly placed onto the thorax of the subjects, who were asked to lay in a supine position. Four ECG electrodes were attached to the skin of the subjects and connected to the device by cables. The signals from patients #CP-01 to #CP-70 and #UP-01 to #UP-21 were acquired at 256 Hz sampling frequency, while those from patients #UP-22 to #UP-30 were sampled at 512 Hz. All the measurements were carried out during quiet breathing.

The SCG signals from the described database were first explored by visual inspection, and only their dorso-ventral components, corresponding to the z-axis acceleration signals, were taken into account. Particularly, 22 SCG recordings characterized by poor signal quality were excluded from the analysis, as they did not allow a clear identification of the heartbeat waveform. A further SCG signal was also excluded since no ECG recordings had been simultaneously acquired. Therefore, a total of 77 SCG and ECG signals were included in this study. Table 1 reports the patient IDs of discarded SCG signals, along with the reasons for their exclusion. 

### 2.2. Signals Pre-Processing

**Figure 1:**
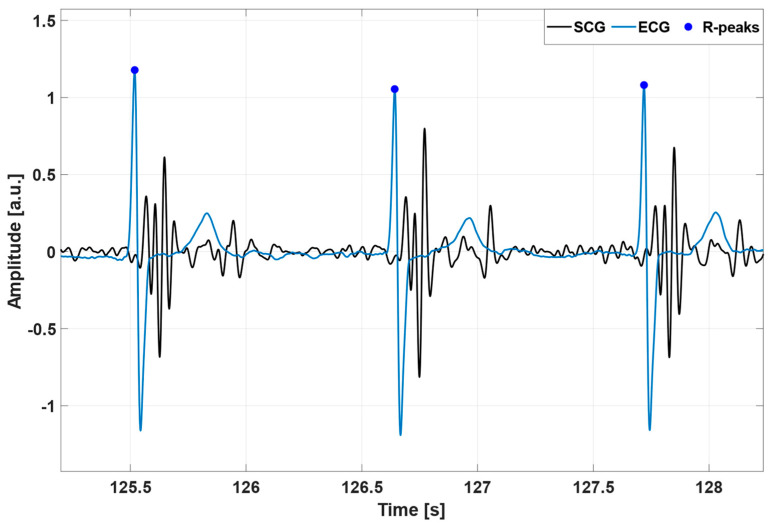
An excerpt ofthe pre-processed SCG (black line) and ECG (blue line) signals from subject #UP-21. The blue points mark the locations of the R-peaks.

All the processing operations described in this study were carried out in MATLAB^®^ R2018b (MathWorks, Inc., Natick, MA, USA). To improve the temporal resolution, the SCG and ECG signals were first oversampled at 1 kHz via linear interpolation by using the MATLAB^®^ function “*interp1*”. Afterwards, the high-frequency component of the SCG signal was extracted via a 4th order zero-lag Butterworth band-pass filter with cut-off frequencies of 7 and 30 Hz. This component has been shown to feature short, oscillatory patterns corresponding to heartbeats [51]. The ECG lead II signal, instead, was first band-pass filtered in the 0.5–40 Hz frequency band via a 4th order zero-lag Butterworth filter. Then, a notch filter was used to remove the 50 Hz powerline interference and its higher harmonics. Finally, the R-peaks were located by means of the well-known Pan and Thompkins algorithm [22], implemented in the “*BioSigKit*” MATLAB^®^ toolbox [83]. An example of the SCG and ECG signals obtained by the pre-processing operations is depicted in Figure 1.

### 2.3. Template Matching

After pre-processing, a template matching technique, which had already been used in an FCG study [79], was applied to the SCG signals for heartbeat detection. This technique consists of three steps: (1) selection of a heartbeat template in the SCG signal; (2) computation of the normalized cross-correlation (NCC) function between the selected template and the SCG signal chunks; and (3) localization of the NCC peaks.

#### 2.3.1. Template Selection

**Figure 2:**
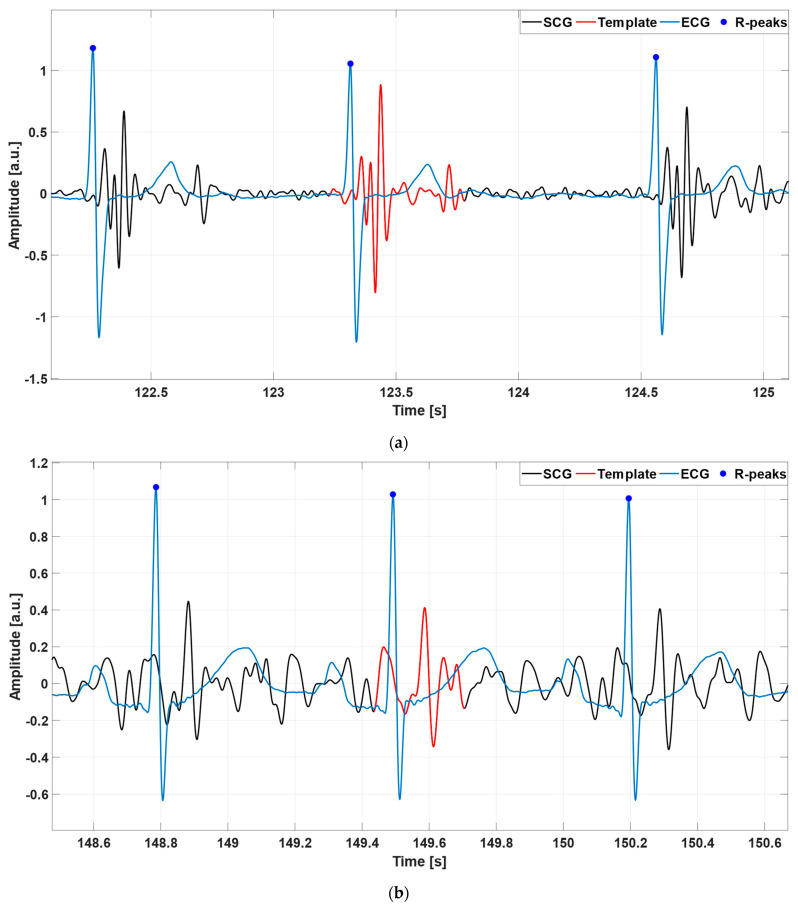
Some examples of the ECG signals (blue lines), SCG signals (black lines), and the selected templates (red lines) from: (**a**) subject #UP-21; (**b**) subject #UP-11. The template in panel (**a**) comprises both the systolic and diastolic complexes in the cardiac cycle, while the one in panel (**b**) includes only the systolic complex.

The template was defined by selecting a single heartbeat in the SCG signal, which was assumed as a morphological reference. For most of the signals, the typical template waveform included both the systolic and diastolic complexes in the cardiac cycle. This was referred to as “case 1”. Figure 2a shows an example of a template comprising both the systolic and diastolic complexes. However, in a number of SCG signals a clear identification of the diastolic complexes was not possible. In this case, referred to as “case 2”, only the systolic complex of the selected heartbeat was eventually included in the template that was employed for heartbeat detection. An example of a template consisting only of the systolic complex is depicted in Figure 2b.

In detail, 50 SCG recordings fell under “case 1”, while the remaining 27 recordings fell under “case 2”. Table 2 reports the template selection criteria adopted in this study, while also showing the template waveforms recommended for both cases. 

#### 2.3.2. Normalized Cross-Correlation and Peaks Localization

**Figure 3:**
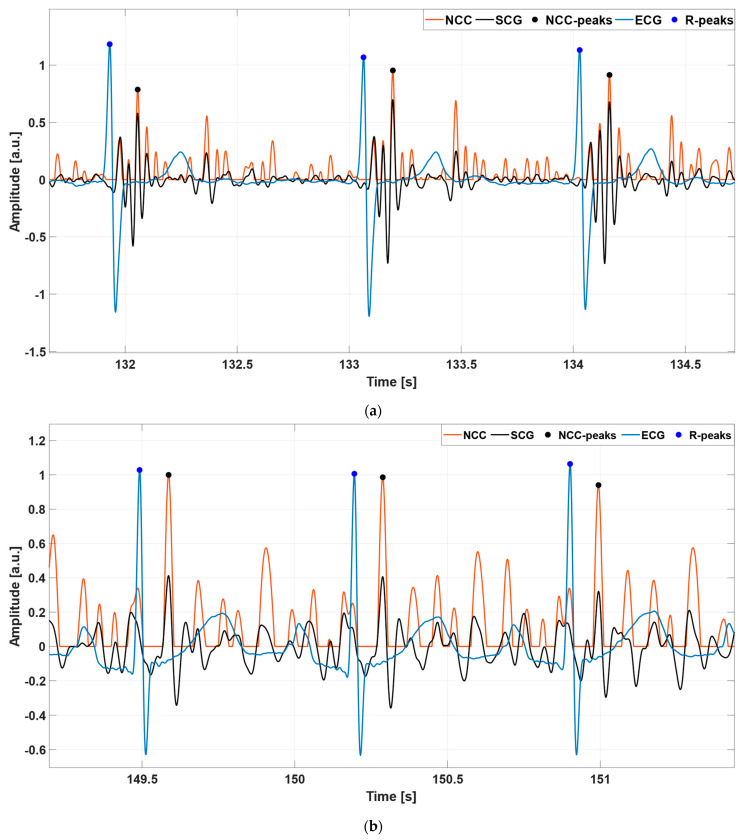
Some excerpts of the ECG (blue line), SCG (black line), and NCC (orange line) signals from: (**a**) subject #UP-21; (**b**) subject #UP-11. The blue and black points mark the locations of the R-peaks and NCC peaks, respectively. The NCC signal in panel (**a**) was obtained by considering the template depicted in Figure 2a, which comprised both the systolic and diastolic complexes in the selected cardiac cycle. The NCC signal in panel (**b**), instead, was obtained by considering the template in Figure 2b, which consisted only of the systolic complex of the selected heartbeat.

After the template had been selected, the NCC function between the SCG signal and the template was computed to evaluate their morphological similarity. The NCC function corresponds to the scalar product between two vectors in the Euclidean space divided by the product of their norms, and is defined by the following mathematical expression:

(1) $$NCC\left[k\right]=\frac{\sum_{n}\left(s\left[n\right]-\mu_{s_{k}}\right)\cdot \left(t\left[n-k\right]-\mu_{t}\right)}{\sqrt{\sum_{n}{\left(s\left[n\right]-\mu_{s_{k}}\right)}^{2}\cdot \sum_{n}{\left(t\left[n\right]-\mu_{t}\right)}^{2}}},$$

where s is the SCG signal, t is the selected template, $\mu_{t}$ is the mean of the template, and $\mu_{s_{k}}$ is the mean of the SCG signal over the shifted template interval. The NCC function has local maxima where the SCG signal reaches the highest similarity with the template. As the template captures the heartbeat pattern in the SCG signal, the highest NCC values occur periodically with the heartbeats. Therefore, the temporal locations of the NCC peaks, which correspond to the heartbeats, were identified via the MATLAB^®^ function “*findpeaks*”. Specifically, a minimum peak prominence of 0.5 was set for the SCG signals characterized by templates of case 1, while a value in the 0.3–0.7 range was considered for the SCG signals with templates of case 2. These values were selected empirically to fit almost all cases, with the aim to reduce as much as possible the need for parameter optimization by the user. Indeed, the same minimum peak prominence of 0.5 chosen for template 1 was used for 50 out of the 77 subjects. In addition, a minimum peak distance of 500 ms was fixed for all signals. Figure 3 shows some examples of the NCC peak detection. In particular, the NCC signal in panel (a) was obtained by considering a case 1 template, which comprised both the systolic and diastolic complexes in the selected cardiac cycle. The NCC signal in panel (b), instead, was obtained by selecting a case 2 template, which consisted only of the systolic complex.

#### 2.3.3. Inter-Beat Intervals Estimation

**Figure 4:**
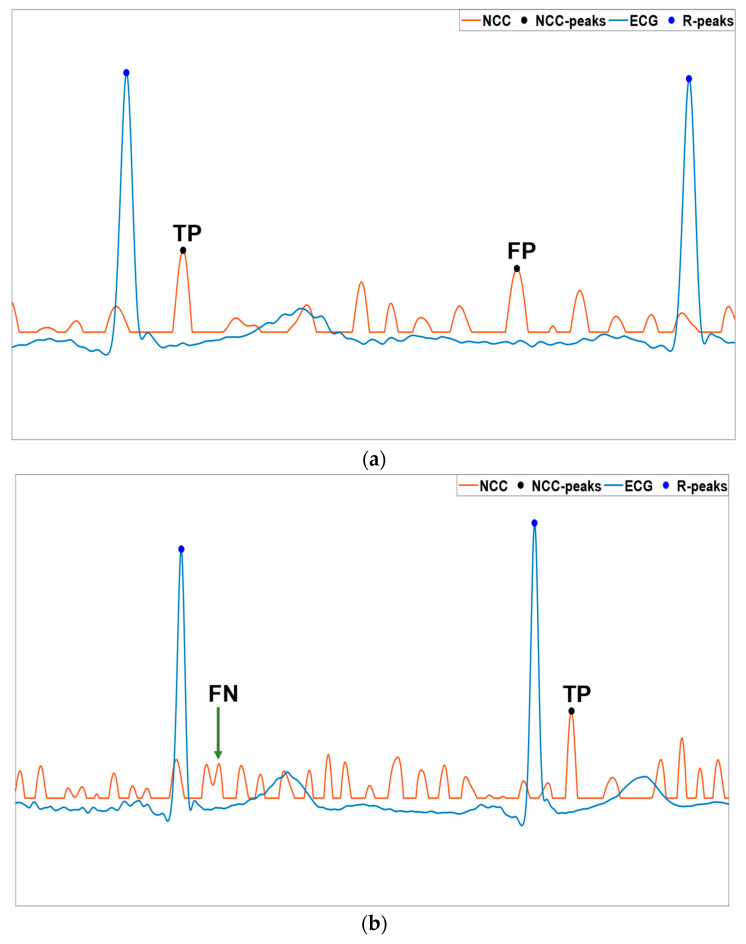

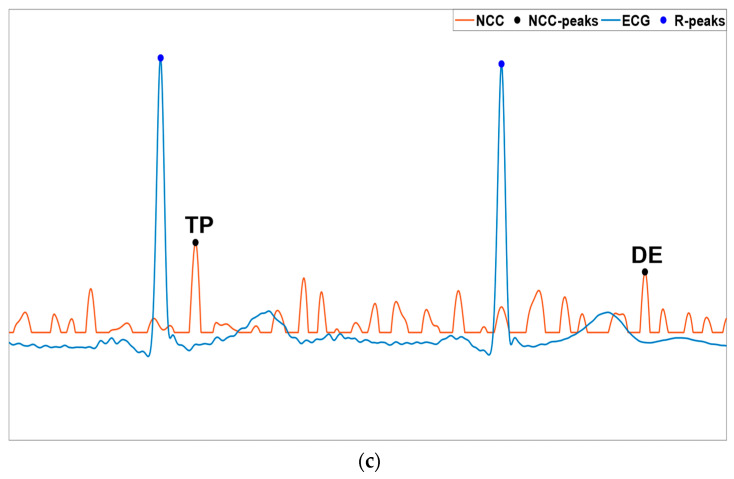
An example of the NCC peak detection from subject #CP-36: (**a**) false positive; (**b**) false negative; (**c**) detection error.

**Figure 5:**
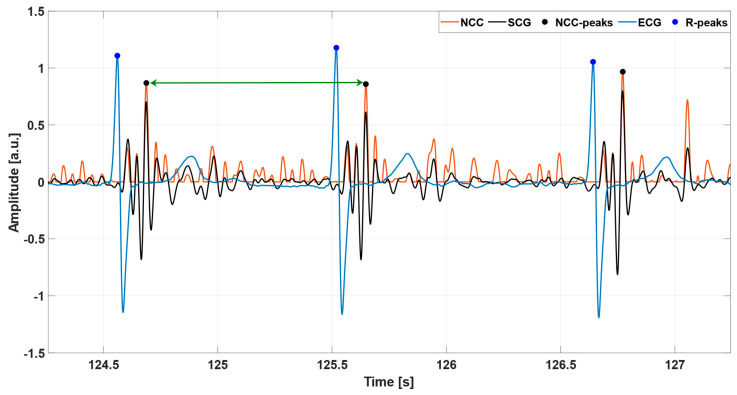
An example of the ECG (blue line), SCG (black line), and NCC (orange line) signals from subject #UP-21. The blue and black points mark the locations of the R-peaks and NCC peaks, respectively. The temporal distance between two NCC peaks (double arrow green line) is referred to as the inter-beat interval.

Once the NCC peaks had been located, the false positives (FP), false negatives (FN), and detection errors (DE) were identified with respect to the actual heartbeats, provided by the reference ECG signals. The false positives were defined as additional NCC peaks within a single cardiac cycle that were wrongly identified as actual heartbeats (see Figure 4a). The false negatives, instead, were considered as actual heartbeats that were missed by the template matching technique (see Figure 4b). The detection errors were referred to as single NCC peaks occurring in a cardiac cycle, but in a temporal location that was not likely to correspond to a matched heartbeat template (see Figure 4c). For this reason, the detection error represented both a false positive, because it marked the presence of a false heartbeat, and a false negative, because no other peaks were found in the same cardiac cycle that marked the presence of the actual heartbeat. Therefore, the number of detection errors contributed both to the numbers of false negatives and false positives. It is noteworthy to underline that all the NCC peaks corresponding to the ECG heartbeats corrupted by motion artifacts were discarded before FP, FN, and DE recognition. Finally, the inter-beat interval estimates were obtained from the ECG signals as the differences between consecutive R-peaks, and from the SCG signals as the differences between consecutive NCC peaks (see Figure 5).

### 2.4. Statistical Analyses

To evaluate the performance of the heartbeat detection using the proposed approach, the sensitivity and positive predictive value (PPV) were computed according to the following equations:

(2) $$Sensitivity=\frac{TP}{TP+FN+DE}\cdot 100,$$

(3) $$PPV=\frac{TP}{TP+FP+DE}\cdot 100,$$

where TP is the number of true positives, FN is the number of false negatives, FP is the number of false positives, and DE is the number of detection errors. The sensitivity quantifies the ability to correctly identify actual heartbeats in the NCC signals, by considering the ECG as the reference, while the PPV quantifies the ability to identify the actual heartbeats among all the detected NCC peaks. Specifically, sensitivity is defined as the percentage of NCC peaks corresponding to actual heartbeats that were correctly identified over the sum of the true and missed NCC peaks (it can also be expressed as the number of TP over the number of R-peaks, which represent reference heartbeats). On the other hand, PPV is defined as the percentage of NCC peaks corresponding to actual heartbeats that were correctly identified over the number of all the detected NCC peaks.

Furthermore, the inter-beat interval measurements from the NCC signals were compared with those estimated from the reference ECG signals by means of regression, correlation, and Bland–Altman analyses [84,85]. The statistical analyses were carried out via the MATLAB^®^ function “*bland-altman-and-correlation-plot*” [86]. The inter-beat intervals related to the FN and DE were excluded from the analyses. 

## 3. Results

### 3.1. NCC Signals with Case 1 Templates

**Figure 6:**
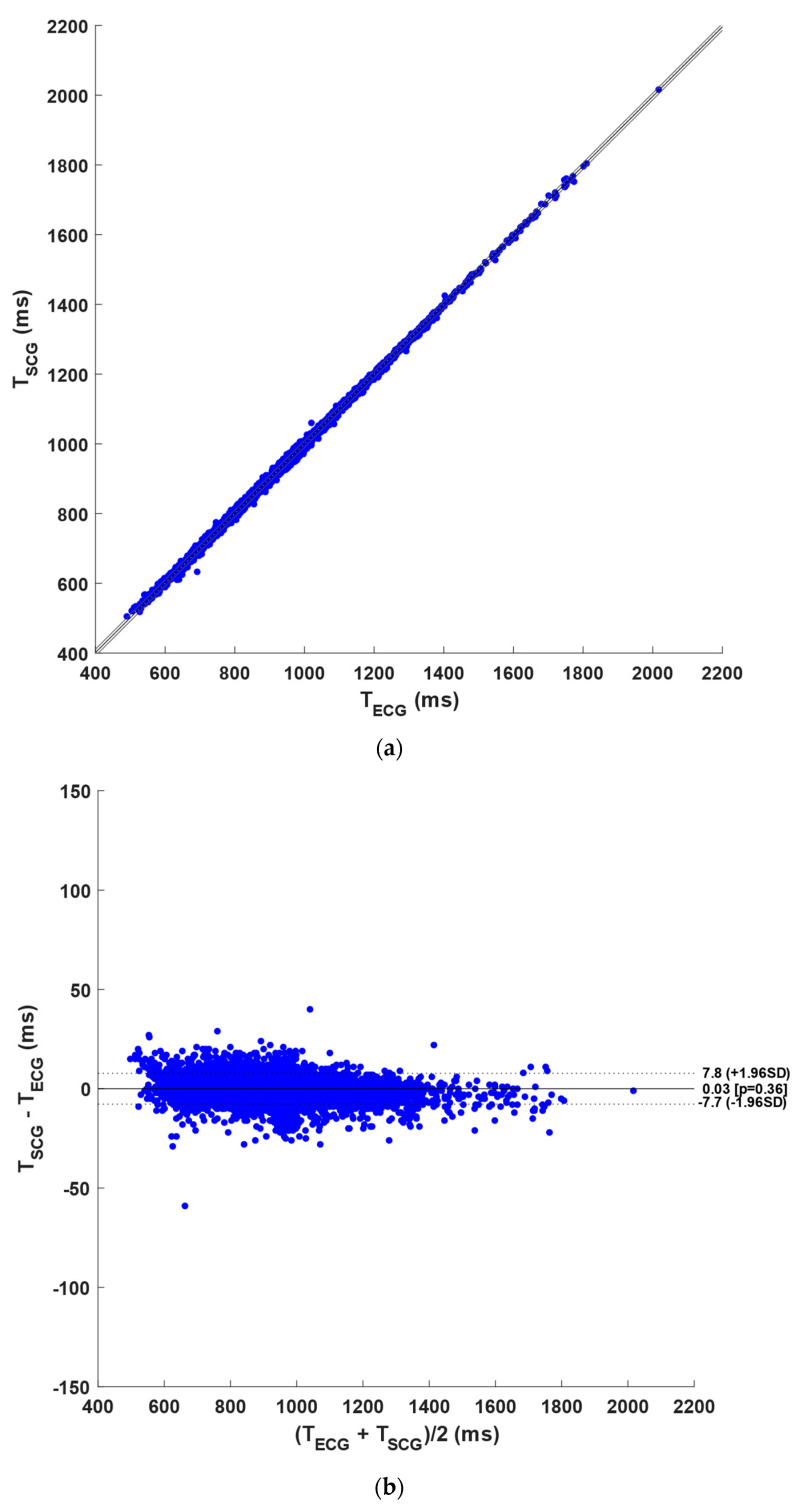
Statistical analyses on the inter-beat intervals: (**a**) results of regression and correlation analyses; (**b**) results of Bland–Altman analysis for the NCC signals with case 1 templates.

Table 3 outlines the number of R-peaks and NCC peaks detected per subject in the ECG and NCC signals with case 1 templates, respectively. The table also reports the number of FPs, FNs, and DEs identified in the NCC signals and the number of inter-beat intervals considered in the statistical analyses. In detail, a total of 19,496 heartbeats were identified in the ECG signals, while 19,318 NCC peaks were detected in the NCC signals, of which 18,741 were TPs, 156 FPs, 334 FNs, and 421 DEs. Therefore, the proposed heartbeat detection approach achieved a sensitivity of 96% and a PPV of 97%. Furthermore, a total of 18,085 inter-beat intervals were compared via the regression, correlation, and Bland–Altman analyses. A slope of 0.997 and an intercept of 2.80 ms, with a R^2^ value greater than 0.999 (*p*-value < 0.0001), were obtained from the regression and correlation analyses (see Figure 6a). Moreover, the Bland–Altman analysis reported a non-significant bias (*p*-value = 0.36), with limits of agreement (LoA) of ±7.8 ms (see Figure 6b). The results of the statistical analyses are also summarized in Table 5.

### 3.2. NCC Signals with Case 2 Templates

**Figure 7:**
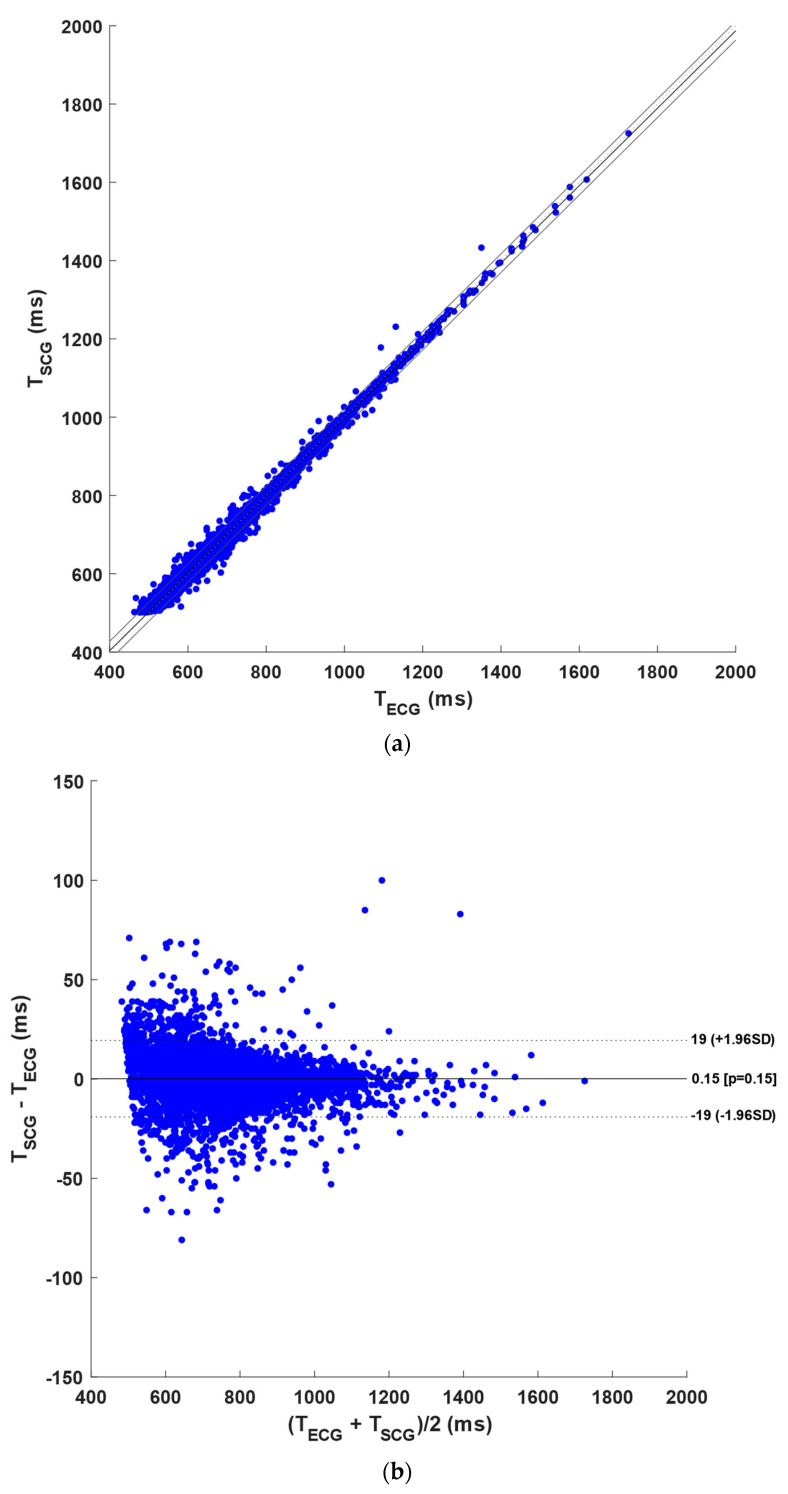
Statistical analyses on the inter-beat intervals: (**a**) results of regression and correlation analyses; (**b**) results of Bland–Altman analysis for the NCC signals with case 2 templates.

Table 4 illustrates the number of R-peaks and NCC peaks detected per subject in the ECG and NCC signals with case 2 templates, respectively. The table also reports the number of FPs, FNs, and DEs in the NCC signals, as compared to the reference ECG, along with the number of compared inter-beat intervals. In detail, a total of 13,769 heartbeats were identified in the ECG signals, while 13,241 NCC peaks, of which 10,842 TPs, 524 FPs, 1049 FNs, and 1875 DEs, were detected in the NCC signals. Hence, the NCC signals scored a sensitivity of 79% and a PPV of 82%. Moreover, the statistical analyses were performed on a total of 8955 inter-beat intervals. The regression and correlation analyses (see Figure 7a) reported a slope of 0.990 and an intercept of 7.62 ms, with a R^2^ value greater than 0.99 (*p*-value < 0.0001). Furthermore, non-significant bias (*p*-value = 0.15) and LoA of ±19 ms resulted from the Bland–Altman analysis (see Figure 7b). The results of the statistical analyses are also outlined in Table 5.

## 4. Discussion

This study described a novel algorithm based on template matching for the detection of heartbeats in SCG signals without the support of concurrent ECG signals. The high inter-subject and intra-subject variability and the noisy nature of SCG signals make the task of ECG-free heartbeat detection rather troublesome. Indeed, the use of ensemble averaging is very common in SCG studies to unveil the underlying waveform corresponding to heartbeats. Unlike previous studies that proposed rather complex algorithms, this study focused on searching for matches between the whole SCG signal and a single heartbeat template selected from the signal itself, by taking advantage of the normalized cross-correlation as a similarity measure with very high robustness to noise.

Two classes of template were used, namely “case 1”, which included both the systolic and diastolic complexes, and “case 2”, which included the systolic complex only. The reason for using the “case 2” template lies in the absence of clear diastolic complexes in the SCG recordings of some patients, which could not be distinguished from the physiological background noise or exhibited significant morphological instabilities that could seriously impair the performance of template matching. According to the results of the statistical analyses, the “case 1” template provided far better performance as compared to the “case 2” template, with a sensitivity of 96% vs. 79%, a PPV of 97% vs. 82%, and limits of agreement of ±7.8 ms vs. ±19 ms, respectively. Indeed, the shapes of individual complexes, both systolic and diastolic, are not very peculiar, so the use of a template that includes only a single complex could lead to detection errors. On the contrary, using a template that includes both systolic and diastolic complexes, which rather represent a pattern that is peculiar to a single heartbeat, is in principle less prone to misdetections, and the experimental results of this study support this hypothesis. 

Considering that the SCG signals analyzed in this study had been acquired from pathological subjects affected by one or more valvular heart diseases, ranging from moderate to severe stages, the results achieved with both the “case 1” and “case 2” templates are surprisingly good, all the more so if the low computational burden of the proposed template matching approach is taken into account. Indeed, the proposed approach essentially requires two simple steps, namely NCC computation and peak detection, which make it also reasonably suitable for real-time implementations without significant computational resource requirements. In light of the promising results obtained at the cost of few processing operations, it seems that the use of artificial intelligence approaches for heartbeat detection in SCG signals, which is undoubtedly an emerging trend in modern research, is not worth its price in terms of the computational burden. It is also important to underline that none of the methods described in the literature have been tested on such a large cohort of pathological subjects as the one considered in this study, therefore their performance has yet to be confirmed on actual patients with cardiac diseases, who certainly represent the actual target of cardiac monitoring.

The main limitation to the proposed approach is the need for the manual selection of the heartbeat template, which makes the overall method operator dependent. However, this study provides qualitative guidelines for template selection, so as to foster the reproducibility of the results. Indeed, a fully automated template selection algorithm would improve the feasibility and reliability of the proposed template matching approach. This would also promote its full implementation in wearable devices for SCG-based cardiac monitoring, as opposed to methods based on artificial intelligence that must rely on cloud computing services and telecommunication infrastructures. While the development of an automated template selection algorithm was out of the scope of this study, it could certainly be the subject of future studies on this topic.

Another limitation to this study is that the SCG signals were all recorded on motionless subjects in supine positions from a single specific point on the chest. It is known that SCG signal morphology depends on body posture and sensor location, and that motion artifacts can seriously hinder signal quality. Therefore, it is very important to assess the performance of the proposed template matching approach in these conditions.

An interesting aspect to be investigated is the suitability of the proposed approach for heart rate variability (HRV) analyses based on SCG. Indeed, Sieciński et al. have already shown in [66] that HRV indices estimated from SCG signals are highly correlated with those extracted from ECG, even in patients with VHDs from the same database considered in this study. However, they determined the heartbeat locations via an algorithm that took advantage of the availability of a simultaneously recorded ECG signal. Therefore, it is important to confirm that the measurement errors achieved by the template matching method proposed in the present study are low enough to ensure the reliable estimation of HRV indices.

Finally, the performance of the proposed approach should be assessed also on a study population that includes patients affected by different cardiac diseases, such as branch blocks, myocardial infarction, cardiac dyssynchrony, and heart failure, because the degree to which such pathologies may undermine the effectiveness of the proposed method is currently unknown and cannot be predicted.

## Figures and Tables

**Table 1:** Patient IDs of discarded SCG signals, along with the reasons for exclusion.

Patient ID #	Reason for Exclusion
CP-03, CP-06, CP-17, CP-18, CP-24, CP-25, CP-29, CP-31, CP-35, CP-46, CP-50, CP-51, CP-54, CP-62, CP-67, UP-02, UP-03, UP-05, UP-19, UP-22, UP-25, UP-26	Poor SCG signal quality
UP-28	Simultaneous ECG signal not acquired

**Table 2:** Template selection criteria. The templates depicted in the table correspond to those highlighted in red in Figure 2.

Case	Description	Typical Template Waveform
1	The template starts from 2–3 oscillations before the systolic peak (highest local maximum), where the amplitude of the oscillations is significantly reduced with respect to the systolic peak amplitude; the template ends just after the last oscillation of the diastolic complex.	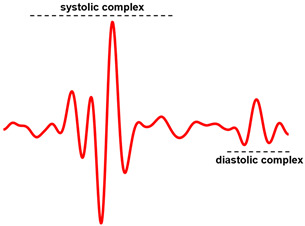
2	The template starts and ends at about 1–2 oscillations before and after the highest systolic peak.	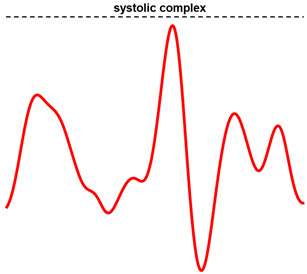

**Table 3:** The number of R-peaks and NCC peaks detected per subject in the ECG and NCC signals by considering case 1 templates, together with the FPs, FNs, and DEs identified in the NCC signals and the number of compared inter-beat intervals.

Patient ID #	R-Peaks	NCC Peaks	FP	FN	DE	Compared Inter-Beat Intervals
CP-01	448	448	0	0	0	447
CP-05	509	507	3	5	8	487
CP-08	544	538	0	6	18	502
CP-12	423	433	14	4	13	388
CP-15	630	623	0	7	3	610
CP-20	391	391	1	1	0	388
CP-21	247	247	2	2	1	241
CP-27	130	130	0	0	0	129
CP-28	238	237	0	1	5	225
CP-30	523	510	0	13	2	498
CP-33	449	456	12	5	12	417
CP-34	462	460	4	6	4	444
CP-36	386	377	8	17	101	166
CP-39	518	518	0	0	8	504
CP-41	349	344	1	6	2	332
CP-44	321	320	2	3	0	314
CP-45	357	343	0	14	17	303
CP-47	537	522	6	21	11	476
CP-49	451	457	11	5	47	353
CP-53	562	561	0	1	0	559
CP-57	507	506	0	1	5	495
CP-58	525	524	0	1	1	520
CP-59	405	406	1	0	0	404
CP-60	512	503	0	9	1	493
CP-61	397	403	6	0	1	395
CP-63	610	608	0	2	0	605
CP-64	382	391	9	0	1	379
CP-65	369	366	0	3	1	360
CP-66	468	468	1	1	0	465
CP-68	327	330	23	20	32	231
CP-69	587	585	0	2	0	582
CP-70	422	412	3	13	33	338
UP-04	258	251	5	12	2	233
UP-06	458	400	1	59	28	312
UP-07	376	373	1	4	2	365
UP-08	257	256	0	1	1	252
UP-09	286	278	0	8	0	273
UP-10	165	157	4	12	2	136
UP-13	106	93	0	13	1	84
UP-14	340	338	1	3	4	325
UP-15	214	189	0	25	0	168
UP-16	221	214	1	8	18	172
UP-18	350	349	0	1	1	345
UP-20	617	615	0	2	2	608
UP-21	305	305	0	0	2	300
UP-23	565	567	2	0	0	564
UP-24	349	340	2	11	9	311
UP-27	269	283	16	2	21	224
UP-29	146	142	0	4	1	136
UP-30	228	244	16	0	0	227
**Total**	**19,496**	**19,318**	**156**	**334**	**421**	**18,085**

**Table 4:** The number of R-peaks and NCC peaks detected per subject in the ECG and NCC signals by considering case 2 templates, together with FPs, FNs, and DEs identified in the NCC signals and the number of compared inter-beat intervals.

Patient ID #	R-Peaks	NCC Peaks	FP	FN	DE	Compared Inter-Beat Intervals
CP-02	651	643	18	26	19	571
CP-04	661	654	1	8	46	556
CP-07	451	453	3	1	5	441
CP-09	364	342	15	37	105	139
CP-10	506	505	63	64	113	213
CP-11	656	662	26	20	56	525
CP-13	837	829	0	8	5	814
CP-14	472	477	36	31	163	157
CP-16	355	360	15	10	13	312
CP-19	484	493	18	9	30	414
CP-22	610	595	16	31	51	466
CP-23	235	225	0	10	28	172
CP-26	389	381	5	13	5	353
CP-32	527	486	38	79	95	225
CP-37	342	335	55	62	90	104
CP-38	406	420	18	4	46	309
CP-40	509	505	26	30	233	114
CP-42	346	340	27	33	41	220
CP-43	460	478	37	19	95	266
CP-48	637	626	16	27	50	500
CP-52	728	532	0	196	38	298
CP-55	793	602	15	206	248	127
CP-56	742	693	15	61	145	409
UP-01	239	241	20	18	31	154
UP-11	417	414	17	20	54	305
UP-12	339	347	23	15	64	208
UP-17	613	603	1	11	6	583
**Total**	**13,769**	**13,241**	**524**	**1049**	**1875**	**8955**

**Table 5:** Results of the statistical analyses. Non-significant bias is indicated as “NS”.

Template Case	Compared Inter-Beat Intervals	R^2^	Slope	Intercept (ms)	Bias	*p*-Value	LoA (ms)	Sensitivity (%)	PPV (%)
1	18,085	>0.999	0.997	2.80	NS	0.36	±7.8	96	97
2	8955	>0.99	0.990	7.62	NS	0.15	±19	79	82

## Data Availability

All relevant research data will be made available upon request after the publication of the paper.
